# Remarks on the Treatment of Jaundice

**Published:** 1840-02-08

**Authors:** James Jackson

**Affiliations:** Late Professor of the Theory and Practice of Medicine at Harvard University; Boston


REMARKS ON THE TREATMENT OF JAUNDICE. By JAMES JACKSON, M. D., late Professor of the Theory and Practice of Medicine at Harvard University.

More than six years ago I had a patient under convalescence from typhoid fever, who became affected with jaundice. With the essential symptoms of that disease, he had a sense of soreness, and a tenderness to the touch, in the right hypochondrium. I directed leeches on this region, and the next day the soreness had gone. At the same time the bile appeared in the alvine dejections, and the skin became less yellow.

All this might be supposed to be a mere coincidence, or to be an effect peculiar to a jaundice connected with inflammation of the liver, or of its peritoneal coat. But as the relief in respect to the jaundice might be explained otherwise, and so as to justify a hope of relief from the same treatment in a simple case of that disease, I was induced to employ the leeches in the next case of the disease which occurred to me.

In this next case the relief was equally decided, and equally prompt.

From that time I have applied leeches in every case of this disease for which I have been consulted. I have not kept an account of these cases, but I think they cannot have been less than twenty. Some of them I have seen in consultation; others were cases in which the ordinary remedies had been administered without success. I can, at this moment, recall to mind eight cases, which have occurred within the last fifteen months. Of the cases I have seen, some had been affected only two or three days, and others three or four weeks when I first visited them. In every case except two, the bile has appeared in the stools within forty-eight hours after the leeches have been applied, notwithstanding the different periods of the disease at which they were applied; and from that time the recovery has proceeded rapidly. Of the two excepted cases, one was a woman whom I saw with one of my medical friends. She had been jaundiced four weeks; and from this disease, and other causes, was much enfeebled. Four leeches, only, were applied. My friend saw something to encourage him, so that we repeated the leeches in a few days,—and then entire relief followed within forty-eight hours. In the other case I applied only six leeches at first. The patient thought himself better, but his stools continued to be destitute of bile. In a few days I directed eight leeches, and then relief followed at once, as the next stool contained bile, and all the symptoms subsided from that day.

Thus, in every case, the bile has resumed its course into the intestines under this treatment, in such a manner as cannot, I think, admit a doubt that it has been in consequence of the treatment.

You will not imagine that I believe the same relief will follow in every case where the skin and eyes are yellow, the urine of a deep colour, and the alvine fæces are destitute of bile. I have heretofore seen cases of this disease, dependent on organic affections, which would not, I am sure, be relieved by leeches; though I have not had any such within the period above specified. Such, for instance, are the cases produced by a scirrhus of the right extremity of the pancreas, involving the excretory duct of this organ, and that of the liver. But in these, and in most cases dependent on organic affections, no remedies can be relied on.

There are few cases of the sort in which leeches would be hurtful; so that one need not be deterred from a trial of them under doubtful circumstances. I ought to add that the treatment may also fail in some cases, which would be relieved by other remedies, though it has not so happened in the limited experience which I have stated to you.

In an adult of common vigour I have usually applied eight or ten foreign leeches, but sometimes only six. In those originally under my own care I have first administered a cathartic, so as to be assured of the colour of the stools.

I do not wish to involve the question of the treatment, which I put on the ground of experience, with any question as to the nature of the disease. Though I have an opinion on this subject, yet it is one not founded on anatomical observation; and that, because the common jaundice is not a fatal disease.

I will only add that I am aware that this disease is relieved under the use of various remedies, and often without the use of any. It is only from the regularity of the amendment, usually shown in the first stool, and always within the period before stated, after one, or at most two applications of leeches, that I am led to attribute that amendment to the remedy.

*Boston*, January 25, 1840.

